# Intraoperative anafilaxis investigation in doctor's office

**DOI:** 10.1186/1939-4551-8-S1-A172

**Published:** 2015-04-08

**Authors:** Ana Carolina Alves Feliciano De Sousa Santos, Ludmilla Luzia Pires Amaral Resende, Celso Taques Saldanha, Lillian Sanchez Lacerda Moraes

**Affiliations:** 1 Universidade De Cuiabá, Brazil; 2 Asbai, Brazil; 3 Universidade Federal De Mato Grosso, Brazil

## Background

Drugs administered during the anaesthetic procedure and postoperative period belong to different pharmacological groups and are to ensure the best possible conditions for surgery and maximum safety of patients. Their adverse side effects are often dependent on immune responses. The diversity and number of the agents administered hinder precise determination of the drug eliciting the adverse drug reaction.

## Methods

Report 3 research cases of anaphylactic reactions during the perioperative period. The skin tests and provocation tests was realized on emergency service in the hospital, consentiment terms was obtained.

## Results

Investigation of 3 adult patients anaphylactic reactions using general anesthesia in underwent surgeries, skin prick test and intradermal was performed with medications used during surgery and medications indicate by anaesthesiologists for next surgery. In case 1, skin prick test and intradermal with cefazolin, propofol, atracurium, fentanyl, morphine and oral provocation with dipirona e ibuprofen was performed, only morphine was positive. In case 2, skin prick test and intradermal with propofol, midazolam, etomidate, atracurium, suxa-methonium, rocuronium, fentanyl, remifentanyl, methadone and tramadol was performed, result atracurium, propofol, midazolam, tramadol and methadone was positive. In case 3, skin prick test and intradermal with propofol, atracurium, fentanyl, midazolam and morphine was performed, result atracurium and fentanyl was positive. The same protocol was used for drugs dilutions. Negative and positive controls was performed. The serum latex IgE was negative. All patients underwent surgery without positive drugs and none had positive reactions during procedure.

## Conclusions

Is possible to investigate intraoperative anaphylaxis outside the university hospital, ensuring patients safety at the next surgery.

